# Pharmacogenetics-based area-under-curve model can predict efficacy and adverse events from axitinib in individual patients with advanced renal cell carcinoma

**DOI:** 10.18632/oncotarget.24715

**Published:** 2018-03-30

**Authors:** Yoshiaki Yamamoto, Ryouichi Tsunedomi, Yusuke Fujita, Toru Otori, Mitsuyoshi Ohba, Yoshihisa Kawai, Hiroshi Hirata, Hiroaki Matsumoto, Jun Haginaka, Shigeo Suzuki, Rajvir Dahiya, Yoshihiko Hamamoto, Kenji Matsuyama, Shoichi Hazama, Hiroaki Nagano, Hideyasu Matsuyama

**Affiliations:** ^1^ Department of Urology, Graduate School of Medicine, Yamaguchi University, Ube, Yamaguchi, Japan; ^2^ Department of Gastroenterological, Breast and Endocrine Surgery, Yamaguchi University Graduate School of Medicine, Ube, Yamaguchi, Japan; ^3^ Department of Computer Science and Systems Engineering, Yamaguchi University Graduate School of Sciences and Technology for Innovation, Ube, Yamaguchi, Japan; ^4^ Faculty of Pharmacy, Kindai University, Higashiosaka, Osaka, Japan; ^5^ Technical Research Laboratory, Toyo Kohan Company Ltd., Kudamatsu, Yamaguchi, Japan; ^6^ School of Pharmacy and Pharmaceutical Sciences, Mukogawa Women's University, Nishinomiya, Hyogo, Japan; ^7^ Department of Laboratory of Analytical Chemistry for Pharmaceutical Sciences, Kindai University, Higashiosaka, Osaka, Japan; ^8^ Department of Urology, San Francisco Veterans Affairs Medical Center and University of California at San Francisco, San Francisco, California, USA; ^9^ Faculty of Pharmacy, Daiichi College of Pharmaceutical Sciences, Fukuoka, Fukuoka, Japan; ^10^ Department of Translational Research and Developmental Therapeutics Against Cancer, Yamaguchi University Faculty of Medicine, Ube, Yamaguchi, Japan

**Keywords:** axitinib, pharmacogenetics, renal cell carcinoma, gene polymorphisms, area under the plasma concentration–time curve

## Abstract

We investigated the relationship between axitinib pharmacogenetics and clinical efficacy/adverse events in advanced renal cell carcinoma (RCC) and established a model to predict clinical efficacy and adverse events using pharmacokinetic and gene polymorphisms related to drug metabolism and efflux in a phase II trial. We prospectively evaluated the area under the plasma concentration–time curve (AUC) of axitinib, objective response rate, and adverse events in 44 consecutive advanced RCC patients treated with axitinib. To establish a model for predicting clinical efficacy and adverse events, polymorphisms in genes including ABC transporters (*ABCB1* and *ABCG2*), *UGT1A*, and *OR2B11* were analyzed by whole-exome sequencing, Sanger sequencing, and DNA microarray. To validate this prediction model, calculated AUC by 6 gene polymorphisms was compared with actual AUC in 16 additional consecutive patients prospectively. Actual AUC significantly correlated with the objective response rate (*P* = 0.0002) and adverse events (hand-foot syndrome, *P* = 0.0055; and hypothyroidism, *P* = 0.0381). Calculated AUC significantly correlated with actual AUC (*P* < 0.0001), and correctly predicted objective response rate (*P* = 0.0044) as well as adverse events (*P* = 0.0191 and 0.0082, respectively). In the validation study, calculated AUC prior to axitinib treatment precisely predicted actual AUC after axitinib treatment (*P* = 0.0066). Our pharmacogenetics-based AUC prediction model may determine the optimal initial dose of axitinib, and thus facilitate better treatment of patients with advanced RCC.

## INTRODUCTION

Axitinib is a highly selective inhibitor of vascular endothelial growth factor receptor (VEGFR) tyrosine kinases 1–3 by blocking downstream signaling through Akt and extracellular signal-regulated kinase (ERK) in renal cell carcinoma (RCC) [1]. Cytochrome P450 (CYP) 3A and uridine diphosphate glucuronosyltransferase (UGT) 1A1 are involved in axitinib metabolism [2, 3]. ATP-binding cassette (ABC) transporters, such as breast cancer resistance protein (BCRP, encoded by *ABCG2*) and P-glycoprotein (MDR1, encoded by *ABCB1*) are efflux transporters expressed on the luminal membranes of enterocytes, and are engaged in the absorption and excretion of several drugs [4]. Previous reports have demonstrated that specific polymorphisms of *ABCG2* and *ABCB1* affect plasma concentrations of tyrosine kinase inhibitors (TKIs), including axitinib [5–7]. These polymorphisms may alter axitinib pharmacokinetics and contribute to determining its optimal dose [8].

Favorable efficacy with acceptable tolerance has been shown in Japanese patients treated with axitinib as first- and second-line therapy for metastatic RCC [9, 10]. In a randomized phase II trial in patients with metastatic RCC, axitinib dose titration was associated with a significantly higher objective response rate (ORR) compared with placebo titration [11]. An observed association between diastolic blood pressure and increased efficacy suggests the potential use of this parameter as a prognostic biomarker [2, 12]. Even where dose titration is an acceptable approach to optimal dose determination, however, the area under the plasma concentration–time curve (AUC) of axitinib [13] for a substantial portion of patients remained low. Another concern may be ethical implications of balancing optimal dosing against the potential for harmful adverse events (AEs) which may increase the risk of cerebrovascular disease [8, 14].

We hypothesized that plasma axitinib concentration and its pharmacokinetic (PK) parameters, including AUC, may correlate with clinical efficacy/AEs, and that the PK data may individually predict these outcomes using baseline patient background. Based on our hypotheses, we established a model to predict clinical efficacy and AEs using polymorphisms in genes that may be related to drug metabolism and efflux. To the best of our knowledge, this is the first report of a pharmacogenomics-based, validated predictive model of axitinib outcomes.

## RESULTS

### Overview

**Figure 1:**
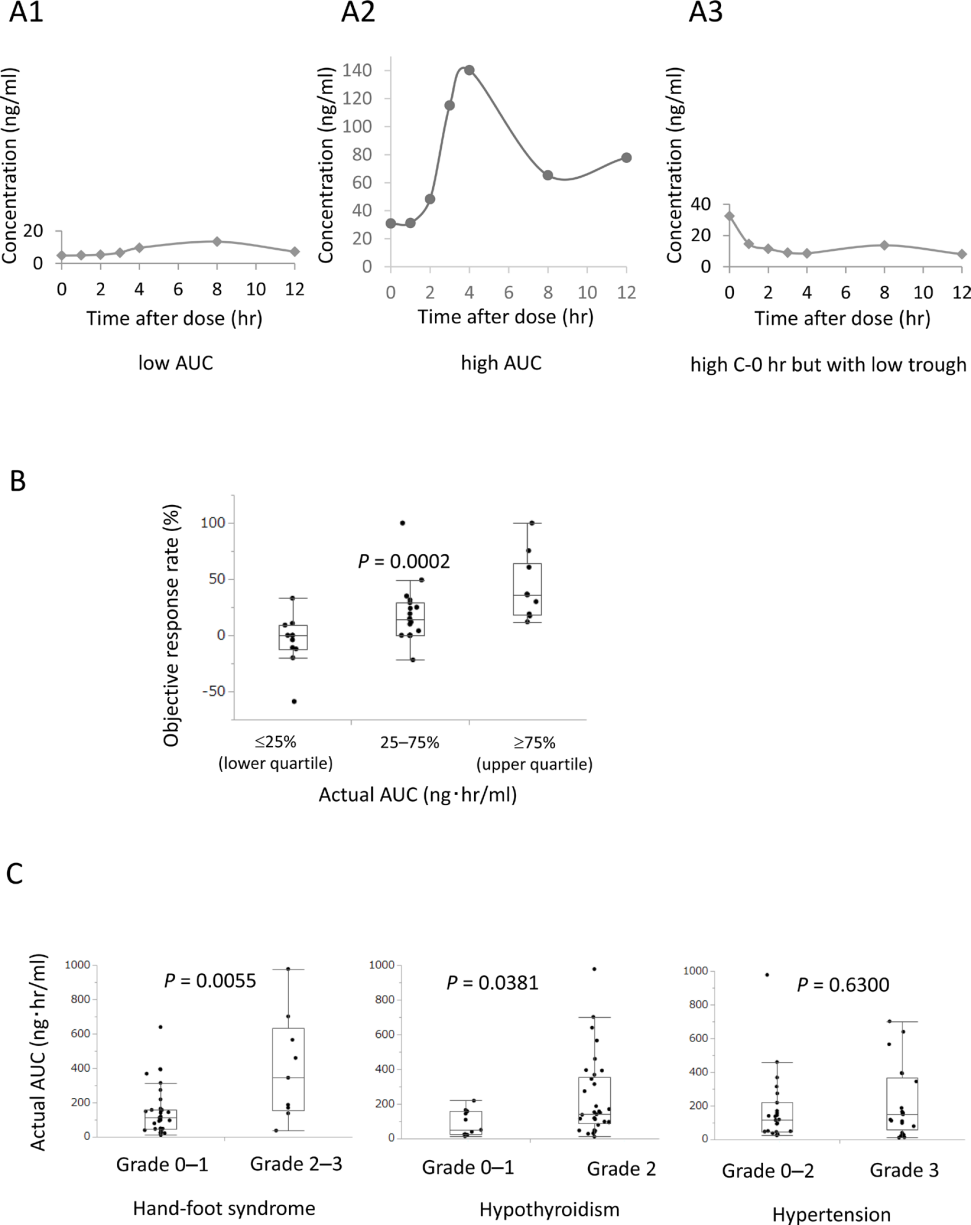
Actual AUC significantly correlated with objective response rate (ORR) and adverse events (AEs) Representative axitinib plasma pharmacokinetics. (**A1**) Representative case with low AUC and C-max and high total clearance. (**A2**) Representative case with high AUC and C-max and low total clearance. (**A3**) Representative case with high C-0 hr but with low trough. (**B**) Patients with higher actual AUC had significantly higher ORR than those with lower actual AUC (*P* = 0.0002). (**C**) Actual AUC significantly correlated with grade 2–3 hand-foot syndrome (*P* = 0.0055) and grade 2 hypothyroidism (*P* = 0.0381), but did not correlate with hypertension (*P* = 0.6300) in AEs.

The individual values for axitinib AUC, total clearance, C-max, C-0 hr, and trough are summarized in Table 1. Plasma concentrations of axitinib differed between individuals (Figure 1A1 and 1A2). C-0 hr was not always consistent with trough value (Figure 1A3). Clinical efficacy and AEs for axitinib in the initial 44 patients are summarized in Table 2.

**Table 1:** Baseline patient characteristics and axitinib plasma pharmacokinetics

Factor	Category	*n* = 60
Age, year	Mean (range)	67.3 (42–90)
Gender	Male/Female	42/18
Prior systemic therapy	Median (range)	16 (1–3)
Pathology	Clear/Non-clear	50/10
Axitinib dose, mg/day	Median (range)	10 (2–12)
ECOG PS	0/1/2–3	46/7/7
AUC, ng, hr/ml	Median (range)	154.5 (11.5–1933.4)
Total Clearance, L/hr	Median (range)	56.2 (5.2–900.9)
C-max, ng/ml	Median (range)	23.3 (1.6–200.8)
C-0 hr, ng/ml	Median (range)	9.7 (0–137.1)
Trough, ng/ml	Median (range)	4.6 (0–86.3)

ECOG PS, Eastern Cooperative Oncology Group performance status.

AUC, area under the plasma concentration–time curve.

Total Clearance, dose/AUC.

C-max, maximal observed plasma concentration.

C-0 hr, observed plasma concentration just before administration.

Trough, trough observed plasma concentration.

**Table 2:** Summary of efficacy and adverse events

Efficacy			*n* = 44	
Best response rate			(%)	
Complete response		1	(2.3)	
Partial response		11	(25.0)	
Stable disease		25	(56.8)	
Progressive disease		3	(6.8)	
Not evaluated		4	(9.1)	
% Best tumor reduction				
Median (range)		13.1	(−58.9–100)	
**Adverse events**			***n* = 44**	
	**G2**	**(%)**	**G3**	**(%)**
Thrombocytpenia	1	(2.3)	0	(0)
Creatinine increased	5	(11.4)	0	(0)
Hypothyroid	33	(75.0)	0	(0)
AST/ALT increased	3	(6.8)	1	(2.3)
Diarrhea	6	(13.6)	2	(4.5)
Hand–foot syndrome	6	(13.6)	3	(6.8)
Proteinuria	10	(22.7)	7	(15.9)
Hypertension	10	(22.7)	21	(47.7)
Fatigue	2	(4.5)	1	(2.3)
WBC decreased	1	(2.3)	0	(0)
Mucositis oral	2	(4.5)	0	(0)

### Actual AUC significantly correlated with ORR and AEs

Patients with higher actual AUC had a significantly higher ORR than those with lower actual AUC (*P* = 0.0002, Figure 1B). A positive correlation between ORR and actual AUC, total clearance, C-0 hr, and trough was found in the linear regression analysis (*P* = 0.0198, 0.0013, 0.0076, and 0.0110, respectively).

Regarding AEs, actual AUC significantly correlated with hand-foot syndrome (*P* = 0.0055) and hypothyroidism (*P* = 0.0381), but not with other AEs including hypertension (*P* = 0.6300, Figure 1C). ORR was associated with hand-foot syndrome (*P* = 0.0147) and hypothyroidism (*P* = 0.0031), but not with hypertension (*P* = 0.6537).

### Pharmacogenetics-based AUC model

**Figure 2:**
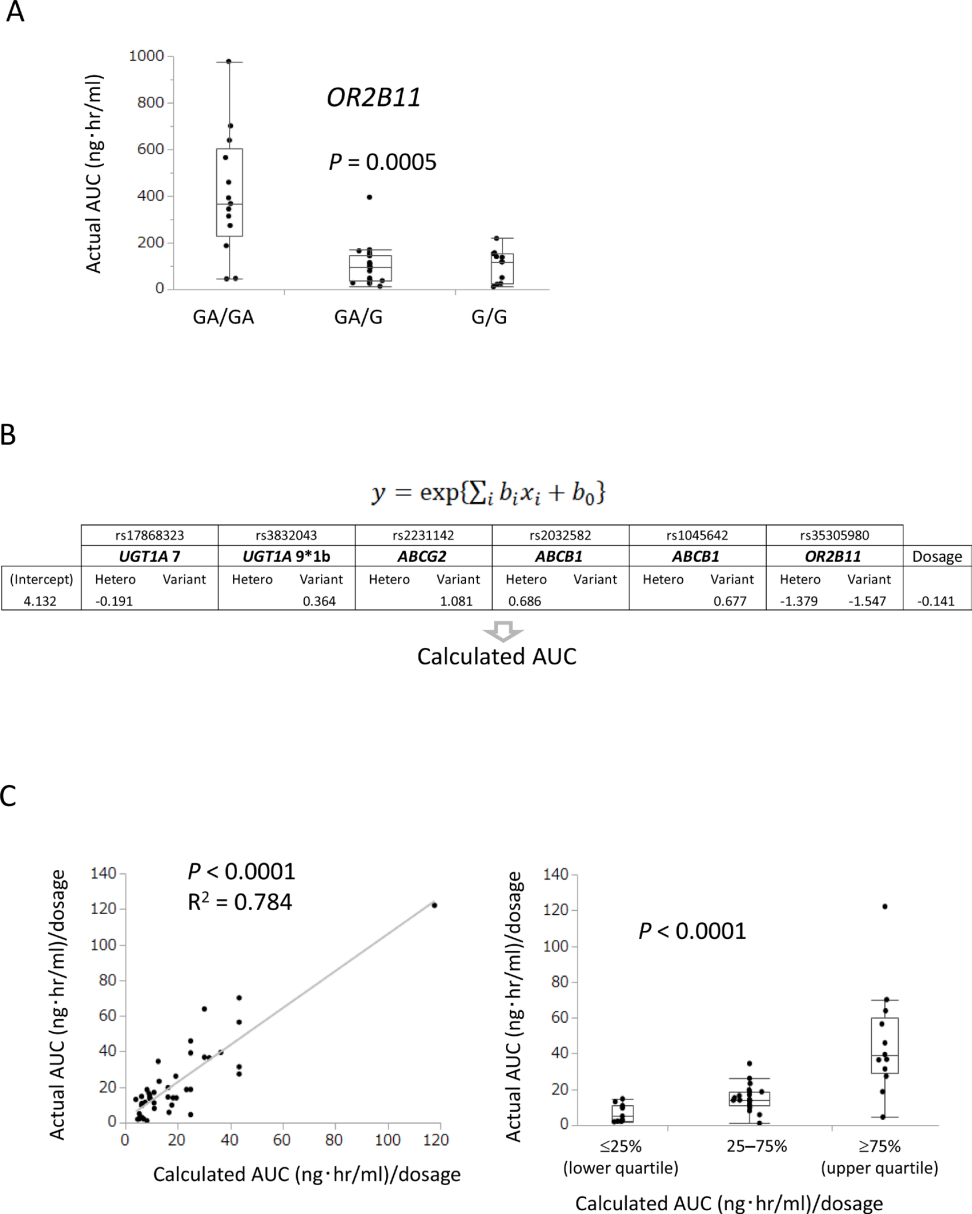
A model to predict clinical efficacy and adverse events was established using gene polymorphisms, including ABC transporters (*ABCB1* and *ABCG2*), *UGT1A*, and *OR2B11* (**A**) *OR2B11* polymorphism identified by whole-exome sequencing significantly correlated with actual AUC in Kruskal–Wallis analysis (*P* = 0.0005). (**B**) Prediction model for AUC using exponential regression with gene polymorphisms and dosage as covariates. (**C**) Positive correlation between calculated AUC and actual AUC was observed in linear regression analysis (*R*^2^ = 0.784, *P* < 0.0001) and Kruskal–Wallis analysis (*P* < 0.0001).

Whole-exome sequencing for germline DNA variants demonstrated that the *OR2B11* variant significantly correlated with actual AUC (*P* = 0.0005, Figure 2A). Figure 2B shows the regression model formula by gene polymorphisms of ABC transporters (ABCB1 and ABCG2), CYP3A, UGT1A, and OR2B11 and coefficient of covariates (parameters of the six genes and axitinib dosage). A statistically significant correlation between calculated AUC and actual AUC was observed in the linear regression analysis (Figure 2C, *R*^2^ = 0.784, *P* < 0.0001) and Kruskal–Wallis analysis (*P* < 0.0001).

### Calculated AUC correlates with clinical efficacy and AEs

**Figure 3:**
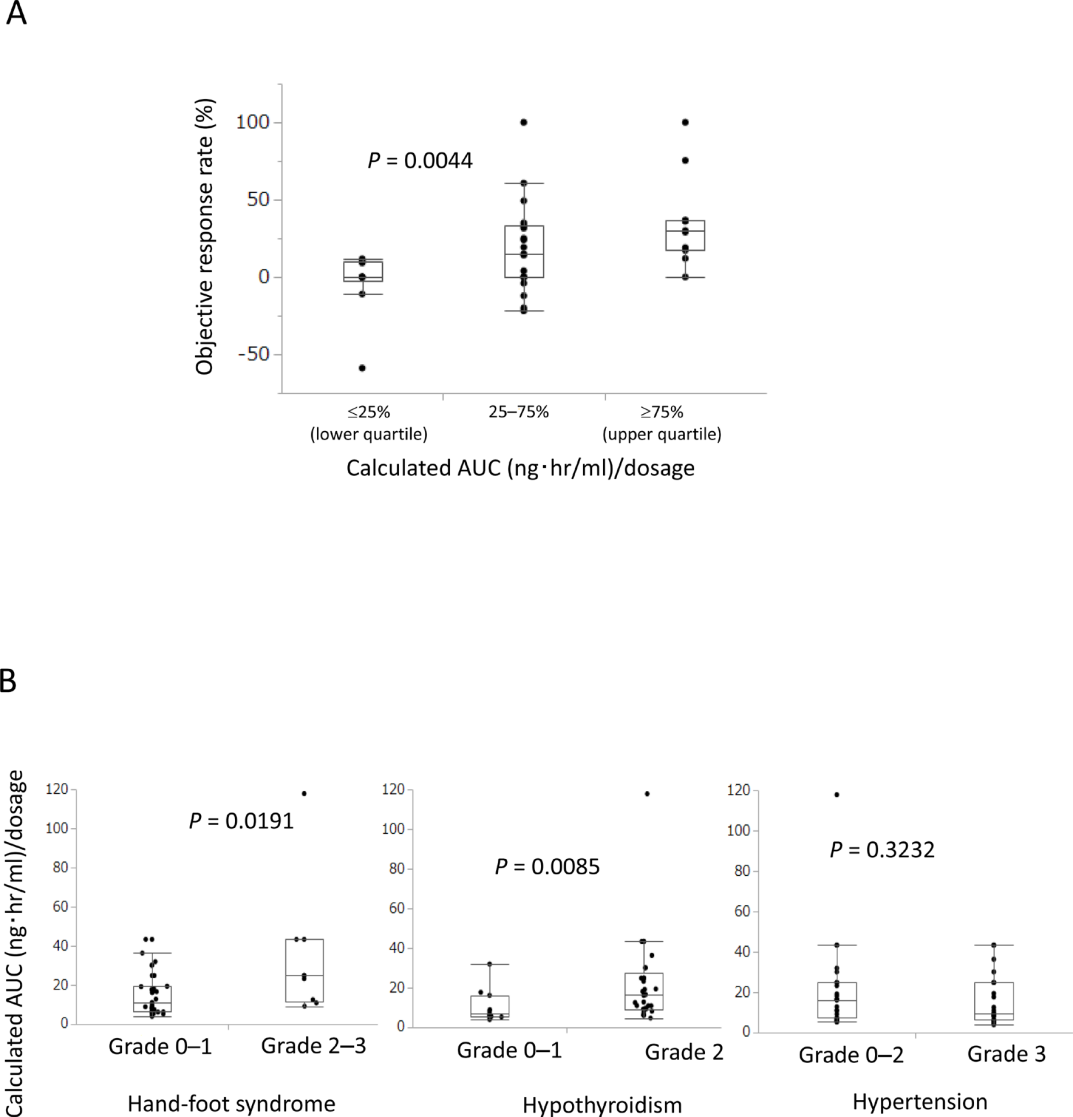
Calculated AUC predicted clinical efficacy and adverse events (AEs) (**A**) Calculated AUC significantly correlated with objective response rate (*P* = 0.0044). (**B**) Calculated AUC significantly correlated with grade 2–3 hand-foot syndrome (*P* = 0.0191) and grade 2 hypothyroidism (*P* = 0.0085), but did not correlate with hypertension (*P* = 0.3232) in AEs.

To validate the model, we evaluated the relationship between calculated AUC and ORR and AEs. Categorized calculated AUC significantly correlated with ORR (*P* = 0.0044, Figure 3A). Calculated AUC also significantly correlated with hand-foot syndrome (*P* = 0.0191) and hypothyroidism (*P* = 0.0085), but not with hypertension (*P* = 0.3232) (Figure 3B).

### Predictive ability of calculated AUC in validation study

**Figure 4:**
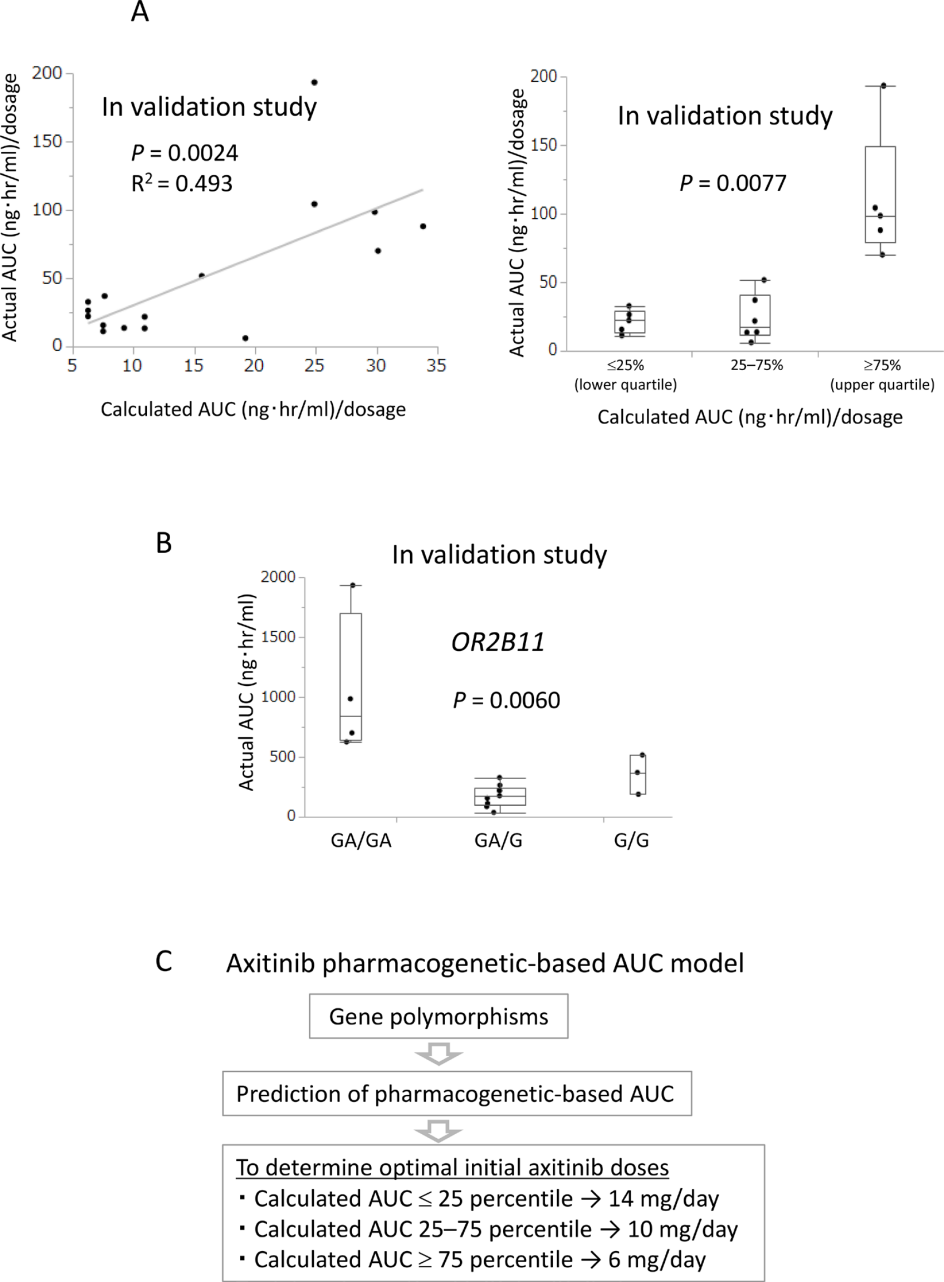
Calculated AUC before axitinib treatment predicted actual AUC after axitinib treatment in the validation study (**A**) In the validation study, a positive correlation between calculated AUC before axitinib treatment and actual AUC after axitinib treatment was found in the linear regression analysis (*R*^2^ = 0.493, *P* = 0.0024) and Kruskal–Wallis analysis (*P* = 0.0077). (**B**) In the validation study, *OR2B11* polymorphism before axitinib treatment significantly correlated with actual AUC after axitinib treatment in Kruskal–Wallis analysis (*P* = 0.0060). (**C**) Axitinib pharmacogenetics-based AUC model to determine optimal initial axitinib doses.

To validate the prediction model, calculated AUC was prospectively compared with actual AUC following axitinib treatment in the 16 additional consecutive patients. A statistically significant correlation was found between calculated AUC before axitinib treatment and actual AUC after axitinib treatment in the linear regression analysis (*R*^2^ = 0.493, *P* = 0.0024) as well as in the Kruskal–Wallis analysis (*P* = 0.0077) (Figure 4A).

## DISCUSSION

Previous studies have demonstrated that higher AUC for axitinib is closely associated with better ORR as well as a more favorable prognosis in metastatic RCC [11, 12]. These reports support our findings that higher AUC is directly associated with better ORR. We demonstrated a close association between higher AUC and higher grade AEs (hypothyroidism and hand-foot syndrome), supporting the reliability of our PK data as these AEs have been reported to be on-target AEs [15]. Beyond our expectations, concentrations of axitinib differed greatly between individuals, up to 100-fold. Wide variations in PK data and our finding that hypertension was associated neither with ORR nor AUC strongly indicate that optimal dosing of axitinib should be determined based on PK data, although 8-point blood sampling (as in our study protocol) for PK data is not realistic in the clinical setting. Our pharmacogenetics-based AUC model, comprising data from several gene polymorphisms, is unique because previous models were based only on a single gene polymorphism associated with drug metabolism and efflux with poor prediction ability [3, 16]. In our model, we selected six polymorphisms, five of which are reportedly associated with axitinib pharmacokinetics and one newly discovered from comprehensive sequencing, and constructed a prediction model of AUC. *OR2B11*, Olfactory Receptor Family 2 Subfamily B Member 11, is related to the olfactory signaling pathway via G-protein-coupled receptors [17]. Although no association has been reported to date between OR2B11 and drug efflux or metabolism, *OR2B11* polymorphism may affect drug efflux through NLRP3 inflammasome-mediated changes in the configuration of the gut microbiota [18]. The inclusion of this gene in our model can be justified by the close association observed between *OR2B11* polymorphism and actual AUC in the validation study (*P* = 0.0060, Figure 4B).

Unexpectedly, hypertension was not associated with ORR. One reason may be the different patient backgrounds, since many patients suffered hypertension and received antihypertensive treatment before axitinib therapy in our study. Concerning other AE, higher AUC was significantly correlated with hypothyroidism. A previous report showed hypothyroidism causes decreasing CYP3A involved in axitinib metabolism [19]. Hypothyroidism derived from axitinib may induce high axitinib AUC. In our study, all (100%) of actual AUC was not consistent with calculated AUC determined by gene polymorphisms. Such mismatched cases may be explained by the fact that the plasma axitinib concentration was also influenced by several factors including age, body weight, smoking, prior systemic therapy, interaction of other drugs [12].

With respect to benefits for patient outcomes, our model is likely to predict the best ORR and several AEs. Best ORR is used as a surrogate biomarker for overall survival in advanced and metastatic RCC [20, 21], while higher AUC did not reach significance to predict progression-free survival (PFS) and overall survival (OS) in a titration study [22, 23]. The lack of an association with PFS and OS in the titration study may be explained by the wide variation in individual AUC values for axitinib treatment, indicating that optimal initial dosing should be determined based on PK data. As our model is based on six gene polymorphisms, optimal initial dosing could be determined prior to axitinib treatment. Dose modification should be considered if the calculated AUC value is at the 25th percentile and less, or at the 75th percentile and more (Figure 4C).

An advantage of DNA microarray technology is that multiple gene polymorphisms can be quickly determined in a single experiment with high accuracy. Therefore, we recommend examining these gene polymorphisms in clinical settings using DNA microarrays to determine the optimal initial dose of axitinib.

In conclusion, our pharmacogenetics-based AUC prediction model may allow the optimal initial axitinib dose to be determined prior to treatment, and might contribute to more precise treatment of individuals with advanced RCC, thus preventing severe AEs.

## MATERIALS AND METHODS

### Study design and patients

A total of 60 patients with histologically diagnosed metastatic or locally-advanced RCC who were treated with axitinib between 2013 and 2017 at Yamaguchi University Hospital were consecutively enrolled in this prospective phase II study (registered to UMIN-CTR; UMIN000011147). To investigate the correlation between PK data and clinical data, plasma concentrations of axitinib were measured and the resulting PK data compared with ORR and AEs reported for the initial 44 consecutive patients. To construct the prediction model for axitinib AUC, we used an exponential regression model with gene polymorphisms and axitinib dosage as covariates. To further validate this prediction model, the calculated AUC was prospectively compared with actual AUC in 16 additional consecutive patients. The characteristics of all 60 patients are summarized in Table 1. This study was approved by the ethical committee of Yamaguchi University Hospital and informed consent was obtained from all patients prior to participation. Axitinib was administered orally at a starting dose of 5 mg twice daily, while dose modification (range: 2–20 mg/day) was conducted based on AEs. Computed tomography was performed every 8 weeks after axitinib initiation. Best ORR and AEs were assessed according to RECIST version 1.1 and CTCAE version 4.0, respectively.

### Plasma concentration of axitinib

Blood samples were collected at 0, 1, 2, 3, 4, 8, and 12 hr after administration of axitinib on day 8. Plasma was separated by centrifugation, stored at −30°C prior to analysis, and analyzed by liquid chromatography with tandem mass spectrometry. The axitinib AUC from 0 to 12 hr was calculated by the trapezoidal method. Total clearance (dose/AUC), maximal plasma concentration (C-max), plasma concentration immediately prior to administration (C-0 hr), and trough plasma concentration (Trough) were also examined. Chromatographic conditions and Mass spectrometric conditions were previously described [24–26].

### Chromatographic conditions

A high-performance liquid chromatography (HPLC) system consisting of an LPG-3400 pump (Thermo Scientific, Tokyo, Japan) with flow control valve, auto sampler (WPS-3000), and temperature controller compartment for the column (TCC-3000) was utilized in this study. Separation was performed in gradient mode with a flow rate of 0.5 ml min^−1^ over a 5-min run time through a reversed phase C18 column. A CAPCELL PAK C18MG II (250 mm × 4.6 mm i.d., Shiseido, Tokyo, Japan) HPLC column was used. The temperature for the auto sampler and column oven was maintained at 10°C and 30°C, respectively. The chromatographic run was performed with a gradient, and the mobile phase consisted of solvent A (HPLC grade water + 0.05% formic acid) and solvent B (HPLC grade acetonitrile + 0.05% formic acid) with a “T” switch tube only 200 μl/min of total flow (0.5 ml/min) introduced into the mass spectrometry (MS) detector.

### Mass spectrometry conditions

MS detection was performed on a Thermo Scientific TSQ Endura system equipped with a heated electrospray ionization (ESI) probe as the ionization source and a triple quadrupole analyzer with positive ionization mode. In the mass spectrometer, nitrogen gas was used as an Aux gas, and sheath gas and ion sweep gas were constantly supplied from a gas generator (System Instruments, Japan). Argon gas was used as a collision-activated dissociation (CAD) gas and was constantly supplied from an argon gas cylinder. MS and MS/MS conditions for pure standards of axitinib were optimized by continuous infusion at 0.5 ml min^−1^ using a syringe pump (Model 11, Harvard Apparatus, Inc., Holliston, MA, USA). The most abundant product ion of each component was selected for the construction of a multiple reaction-monitoring (MRM) method. The transitions monitored were 387.12/356.05 and 488.17/401.12 for AXI and DST, respectively. Aux gas, sheath gas, ion sweep gas, ion spray voltage, and vaporizer temperature were set at 15 psi, 50 psi, 2 psi, 3500 V and 400°C, respectively. The collision energy was set at 22 and 31 eV for AXI and DST, respectively. The mass spectrometer was operated in unit resolution mode for both Q1 and Q3 in the MRM mode. All data were acquired with auto tuning using Quantum tune software (Thermo Scientific).

### Whole-exome sequencing

Lymphocyte DNA was extracted using a QIAamp DNA Mini Kit (Qiagen, Valencia, CA, USA). Whole-exome sequencing was performed in the initial 44 consecutive patients to comprehensively analyze germline DNA variants in the entire exome. DNA quantity was determined with using fluorometric quantitation with a Qubit 3.0 Fluorometer (Thermo Scientific, Tokyo, Japan) as well as by spectrophotometric quantitation using a NanoDrop (Thermo Scientific). DNA quality was examined by agarose gel electrophoresis. A total of 50 ng of genomic DNA for each sample was used to prepare *in vitro* DNA libraries with a Nextera Rapid Capture Exome Kit (Illumina, Tokyo, Japan), producing a total target size of 45 Mb. Sequencing of paired-end fragments (75 bp × 2) was conducted on an Illumina NextSeq 500 sequencing platform (Illumina).

### Data analysis of whole-exome sequencing

The obtained next-generation sequencing data were subjected to reads cleaning with cutadapt (version 1.2.1) (http://cutadapt.readthedocs.io/en/stable/guide.html) and cmpfastq_pe.pl software (http://compbio.brc.iop.kcl.ac.uk/software/cmpfastq_pe.php). After a quality control step, the filtered short reads were mapped to the reference genome (hg19) with BWA (version 0.7.12). The Genome Analysis Tool Kit (GATK; version 3.5) was used to perform local realignment and for the detection of single-nucleotide and insertion/deletion (InDel) polymorphisms. Furthermore, each detected variant was annotated with information such as the genome position and functional effect using SnpEff (version 4.1 k). Annotation of all variants filtered by quality control was then conducted using the Variant Effect Predictor, including annotation with dbSNP146. Following the identification of damaging variants predicted by SnpEff, Sorting Intolerant From Tolerant (SIFT), Polyphen-2, or PROVEAN as damaging, a case-control association analysis was conducted between AUC high and low patients using a trend model (Cochran–Armitage test) in PLINK (version 1.902b3w). The concordance rate between whole-exome sequencing and Sanger sequencing was 99.3%.

### Sanger sequencing

Gene polymorphisms of *ABCB1*, *ABCG2*, *CYP3A*, *UGT1A*, and *OR2B11* were examined by Sanger sequencing. PCR conditions were 95°C for 5 minutes; 40 cycles of 95°C for 30 seconds, 60°C for 30 seconds, and 72°C for 30 seconds; and 72°C for 10 minutes. PCR products were purified using a QIAquick PCR purification kit (Qiagen, Hilden, Germany), cycle-sequenced using a BigDye Terminator version 3.1 cycle sequencing kit (Applied Biosystems; Thermo Fisher Scientific, Inc., Waltham, MA, USA) according to the manufacturer's protocol, and resolved on an ABI 3500 × L sequencer (Applied Biosystems). Sequences were analyzed using Sequence Scanner Software v2.0 (Applied Biosystems). The primers used are shown in Supplementary Table 1.

### DNA microarray

Gene polymorphisms of *ABCB1*, *ABCG2*, *CYP3A*, *UGT1A*, and *OR2B11* were examined by DNA microarray (Gene Chip, Toyo Kohan Co., Kudamatsu, Japan) in parallel with sequencing. A focused DNA microarray was developed on a small chip measuring 3 mm^2^ in size. Primers were labeled with IC5-OSu (N-ethyl-N′-[5-(N′′-succinimidyloxycarbonyl) pentyl]-3,3,30,30-tetramethyl-2,20-indodicarbocyanine iodide; kex = 640 nm and kem = 660 nm; Dojindo Laboratories, Kumamoto, Japan). Multiplex PCR was performed in a 20 μl volume using 0.05 U FastStart Taq DNA polymerase (Roche Diagnostics, Indianapolis, IN, USA) and 20 ng genomic DNA and the following cycle procedure: 40 cycles of denaturation at 95°C for 30 s, annealing at 60°C for 30 s, and elongation at 72°C for 30 s. IC5-labeled DNA was hybridized to probes on the microarray at 55°C for 60 min. The fluorescence intensity (FI) was measured using a Bioshot charge-coupled device camera (Toyo Kohan, Tokyo, Japan). The primers and probes used are shown in Supplementary Table 2. The concordance rate between Sanger sequencing and DNA microarray analysis was 99.7%.

### Pharmacogenetics-based AUC model

We constructed a prediction model of the standard AUC using axitinib dosage data from the initial 44 patients and the following six genotypes: *UGT1A7* (387T>G, rs17868323), UGT1A9*1b (-118T9>T10, rs3832043), *ABCG2* (421C>A, rs2231142), *ABCB1* (2677G>T/A, rs2032582), *ABCB1* (3435C>T, rs1045642), and *OR2B11* (23delT, rs35305980). Genotypes were categorized into three groups: wild-type, hetero, and variant. In our prediction model, the genotypes were expressed as (0, 0), (1, 0), or (0, 1) using two variables (*x_i_*, *x_i+1_*) and dosage was expressed as *x_j_*. We used 8 variables out of 13 candidates to construct the regression model, where $y=\exp\left\{\sum_{i}b_{i}x_{i}+b_{o}\right\}$ represents the predicted standard AUC (AUC/dose, mg/day). We trained the regression model by minimizing the squared error between the logarithm of the standard AUC and $\sum_{i}b_{i}x_{i}+b_{o}$ for 44 patients, where is the actual standard AUC.

### Statistical analysis

Statistical analysis was performed using JMP (version 13) statistical software (SAS Institute, Cary, NC, USA). PK data and ORR as continuous values were tested using linear regression analysis, and categorized data were tested using the Mann–Whitney *U* test or Kruskal–Wallis test. Levels of statistical significance were set at *P* < 0.05. The prediction model was constructed using R freeware (version 2.3.2, R Core Team, Vienna, Austria; https://www.R-project.org).

## SUPPLEMENTARY MATERIALS TABLES


