# Supplementary material for: Pharmacogenetics-based area-under-curve model can predict efficacy and adverse events from axitinib in individual patients with advanced renal cell carcinoma
Source: Oncotarget. 2018 Mar 30;9(24):17160–70. doi: 10.18632/oncotarget.24715 (PMC5908314; doi:10.18632/oncotarget.24715)
Supplement: Supplementary file 1 [file oncotarget-09-17160-s001.pdf]

Pharmacogenetics-based area-under-curve model can predict efficacy and adverse events from axitinib in individual patients with advanced renal cell carcinoma

SUPPLEMENTARY MATERIALS

Supplementary Table 1: Primers used in Sanger sequencing

Reference SNP ID number	Gene Primers	Sequence (5′ to 3′)
rs4148323	UGT1A1*6_FW	CCATGCTGGGAAGATACTGTTGA
	UGT1A1*6_RV	GATCACACGCTGCAGGAAAG
rs8175347	UGT1A1*28_FW	TAGTCGTCCTTCTTCCTCTCTGGT
	UGT1A1*28_RV	ATGGCGCCTTTGCTCCT
rs4124874	UGT1A1*60_FW	AAACCCGGACTTGGCACTT
	UGT1A1*60_RV	CACCTGTCCAAGCTCATTCCT
rs7586110	UGT1A7*12_FW	TCAATGTCGTCAAGGCCAAAA
	UGT1A7*12_RV	GCAAAGCCACAGGTCAGCA
rs17868323	UGT1A7_FW	GATCAGGACCGGGAGTTCA
	UGT1A7_RV	AAAGTCAGTTCGCAACAACCAA
rs3832043	UGT1A9*1b_FW	TGCTCTGGGACAAATTCCAA
	UGT1A9*1b_RV	AGCAGACACACACATAGAGGAAGG
rs10929302	UGT1A1*93_FW	GCACGCAATGAACAGTCATAGTA
	UGT1A1*93_RV	TCCTGGGCACAGAAAATTCAGAGT
rs2740574	CYP3A4*1b_FW	CTGGGTTTGGAAGGATGTGT
	CYP3A4*1b_RV	TGGAGCCATTGGCATAAAAT
rs4986910	CYP3A4*3_FW	TACTAGTTGAGGGGTGGCCC
	CYP3A4*3_RV	AGGAGAAGTTCTGAAGGACTCTGA
rs776746	CYP3A5*3_FW	CCCACGTATGTACCACCCA
	CYP3A5*3_RV	TGTACGACACACAGCAACCT
rs10264272	CYP3A5*6_FW	ATAGCCCACATACTTATTGAGAGAA
	CYP3A5*6_RV	AAGGACGGTAAGAGGTGCTG
rs2231142	ABCG2_BCRP_FW	TGGGTGTACAGATAGGGGGT
	ABCG2_BCRP_RV	TGACCCTGTTAATCCGTTCG
rs1128503	ABCB1_MDR1(1236)_FW	CCTGTGTCTGTGAATTGCCTTG
	ABCB1_MDR1(1236)_RV	TGCATCAGCTGGACTGTTGT
rs2032582	ABCB1_MDR1(2677)_FW	GCAGGCTATAGGTTCCAGGC
	ABCB1_MDR1(2677)_RV	AGTCCAAGAACTGGCTTTGCT
rs1045642	ABCB1_MDR1(3435)_FW	TGAATGTTCAGTGGCTCCGA
	ABCB1_MDR1(3435)_RV	ACAGGAAGTGTGGCCAGATG
rs35305980	OR2B11_FW	TGAGAGCGGTTGGGTGTTAC
	OR2B11_RV	CCTACCTCAAAGGCCAGGTG

Supplementary Table 2: Primers and probes used in DNA microarray analysis

Reference SNP ID number	Gene	Sequence (5' to 3')
Primers		
rs4148323	UGT1A1*6_FW	CCATGCTGGGAAGATACTGTTGA
	UGT1A1*6_RV	GATCACACGCTGCAGGAAAG
rs8175347	UGT1A1*28_FW	TAGTCGTCCTTCTTCCTCTCTGGT
	UGT1A1*28_RV	ATGGCGCCTTTGCTCCT
rs4124874	UGT1A1*60_FW	AAACCCGGACTTGGCACTT
	UGT1A1*60_RV	CACCTGTCCAAGCTCATTCCT
rs7586110	UGT1A7*12_FW	TCAATGTCGTCAAGGCCAAAA
	UGT1A7*12_RV	GCAAAGCCACAGGTCAGCA
rs17868323	UGT1A7_FW	GATCAGGACCGGGAGTTCA
	UGT1A7_RV	AAAGTCAGTTCGCAACAACCAA
rs3832043	UGT1A9*1b_FW	TGCTCTGGGACAAATTCCAA
	UGT1A9*1b_RV	AGCAGACACACACATAGAGGAAGG
rs10929302	UGT1A1*93_FW	GCACGCAATGAACAGTCATAGTA
	UGT1A1*93_RV	TCCTGGGCACAGAAAATTCAGAGT
rs2740574	CYP3A4*1b_FW	CTGGGTTTGGAAGGATGTGT
	CYP3A4*1b_RV	TGGAGCCATTGGCATAAAAT
rs4986910	CYP3A4*3_FW	TACTAGTTGAGGGGTGGCCC
	CYP3A4*3_RV	AGGAGAAGTTCTGAAGGACTCTGA
rs776746	CYP3A5*3_FW	CCCACGTATGTACCACCCA
	CYP3A5*3_RV	TGTACGACACACAGCAACCT
rs10264272	CYP3A5*6_FW	ATAGCCCACATACTTATTGAGAGAA
	CYP3A5*6_RV	AAGGACGGTAAGAGGTGCTG
rs2231142	ABCG2_BCRP_FW	GGATGATGTTGTGATGGGCA
	ABCG2_BCRP_RV	ATCCACACAGGGAAAGTCCT
rs1128503	ABCB1_MDR1(1236)_FW	CCTGTGTCTGTGAATTGCCTTG
	ABCB1_MDR1(1236)_RV	TGCATCAGCTGGACTGTTGT
rs2032582	ABCB1_MDR1(2677)_FW	TGTTGTCTGGACAAGCACTG
	ABCB1_MDR1(2677)_RV	GTCCAAGAACTGGCTTTGCT
rs1045642	ABCB1_MDR1(3435)_FW	GTTTTCAGCTGCTTGATGGC
	ABCB1_MDR1(3435)_RV	GTATGTTGGCCTCCTTTGCT
rs35305980	OR2B11_FW	CATCTGAGGGACTGTCGTGG
	OR2B11_RV	AACACTCTGACGGTGATGGG
Probes		
rs4148323	UGT1A1*6_G	TAAAATGCTCCGTCTCTGATG
	UGT1A1*6_A	TAAAATGCTCTGTCTCTGATGT
rs8175347	UGT1A1*28_TA6	TTTTTGCCATATATATATATATATAAGTAGGA
	UGT1A1*28_TA7	GTTTTTGCCATATATATATATATATATAAGTAGG
rs4124874	UGT1A1*60_T	GCTTTGTTCAAACTGAACTCT
	UGT1A1*60_G	GCTTTGTTCACACTGAACT
rs7586110	UGT1A7*12_T	GTACTTCTTCCACTTACTATATTATAG
	UGT1A7*12_G	TACTTCTTCCACGTACTATATTATA
rs17868323	UGT1A7_T	TACTAATTTTTTGTCCTTAAACAAAC
	UGT1A7_G	CTACTAATTTTCGGTCATTAAACA

rs3832043	UGT1A9*1b_T9	AGTGACTGATTTTTTTTTATGAAAGG
	UGT1A9*1b_T10	GTGACTGATTTTTTTTTTATGAAAG
rs10929302	UGT1A1*93_G	AATGAGCTTGGACAGGTGG
	UGT1A1*93_A	AATGAGCTTAGACAGGTGG
rs2740574	CYP3A4*1b_A	AAGGGCAAGAGAGAG
	CYP3A4*1b_G	AAGGGCAGGAGAGAG
rs4986910	CYP3A4*3_T	CAAACCTCATGCCAATG
	CYP3A4*3_C	CAAACCTCGTGCCAAT
rs776746	CYP3A5*3_A	TGTCTTTCAATATCTCTTC
	CYP3A5*3_G	TGTCTTTCAGTATCTCTTC
rs10264272	CYP3A5*6_G	TAGGAACTTCTTAGTGCTC
	CYP3A5*6_A	TAGGAACTTTTTAGTGCTC
rs2231142	ABCG2_BCRP_C	AGAGAAAACTTACAGTTCTCAGCA
	ABCG2_BCRP_A	AGAGAAAACTTAAAGTTCTCAGCAG
rs1128503	ABCB1_MDR1(1236)_T	TTGAAGGGTCTGAACCT
	ABCB1_MDR1(1236)_C	TTGAAGGGCCTGAACCT
rs2032582	ABCB1_MDR1(2677)_G	CTAGAAGGTGCTGGGAAGGT
	ABCB1_MDR1(2677)_T	AACTAGAAGGTTCTGGGAAGGTG
	ABCB1_MDR1(2677)_A	AACTAGAAGGTACTGGGAAGGTG
rs1045642	ABCB1_MDR1(3435)_C	AAGAGATCGTGAGGGCAG
	ABCB1_MDR1(3435)_T	GAAGAGATTGTGAGGGCA
rs35305980	OR2B11_GA	GAGTCCCCTAAGAAGCTATGGTTG
	OR2B11_G	GAGTCCCCTAAGAGCTATGGTTG
